# The natural time course of myocardial oedema in the 12 months post ST-elevation MI in patients treated with primary angioplasty

**DOI:** 10.1186/1532-429X-14-S1-P32

**Published:** 2012-02-01

**Authors:** Thomas R Burchell, Mark Westwood, Steffen E Petersen, Saidi A Mohiddin, Ceri Davies

**Affiliations:** 1 Cardiology, The London Chest Hospital, London, UK

## Background

Myocardial oedema, myocardial salvage and myocardial salvage index, have been described as markers of prognosis and surrogate markers for clinical trials. The time-course of oedema following revascularised MI has been described in the first 6 months following PPCI but there is wide disagreement on the time to complete resolution. We therefore aimed to determine the time-course of post infarct myocardial oedema using serial T2-weighted CMR imaging for the first year following MI.

## Methods

16 patients with acute ST-elevation MI who underwent primary PCI with stent implantation within 12 hours of symptom onset were recruited. Patients were scanned on days 1, 3, 10, 20, 96 and 384 days following their PPCI with a 1.5T Philips Achieva (Philips Medical Systems). Images were obtained as continuous short-axis stacks covering the left ventricle with a slice thickness of 8mm and gap of 2mm. Myocardial oedema was assessed at all time points using T2-weighted triple inversion turbo spin echo STIR imaging (TE 80, TR 1667). Image analysis was performed using dedicated software, CMR42 (Circle CVI, Calgary, Canada). Scar and oedema volumes were calculated by manually drawing endocardial and epicardial contours followed by semi-automated selection of normal remote myocardium per slice. The oedema was described as >2SD in signal intensity from remote normal myocardium. Values are expressed as a percentage of the LV mass (%LVM).

## Results

Patient age was 55.6 ± 8.7 years (94% male). Myocardial oedema was identified in all patients on days 1, 3, 10 and 20 post MI and on day 96 it was present in all but two of the 16 patients. On day 384 the oedema had resolved in 15 of the 16 patients, with one patient still demonstrating myocardial oedema in the same coronary territory (this patient had recently had a further NSTEMI prior to this last scan). A repeated measures 1-way Anova showed a significant difference between the means (p=<0.0001). A Bonferroni's Multiple Comparison Test was performed, showing no significant difference between days [1 and 3], [1 and 10] and [10 and 20]. All other comparisons were significant (p=<0.001). A post-test for linear trend showed a downward slope of -3.24 (p=<0.0001).

## Conclusions

Myocardial oedema post successfully revascularised ST-elevation MI peaks at day 3, persists at 96 days and shows complete resolution at 1 year. There is no significant change between days 10-20 which may provide the most appropriate time period to assess myocardial oedema.

## Funding

This study was funded by an unrestricted grant from Barts and The London Charity.

**Figure 1:**
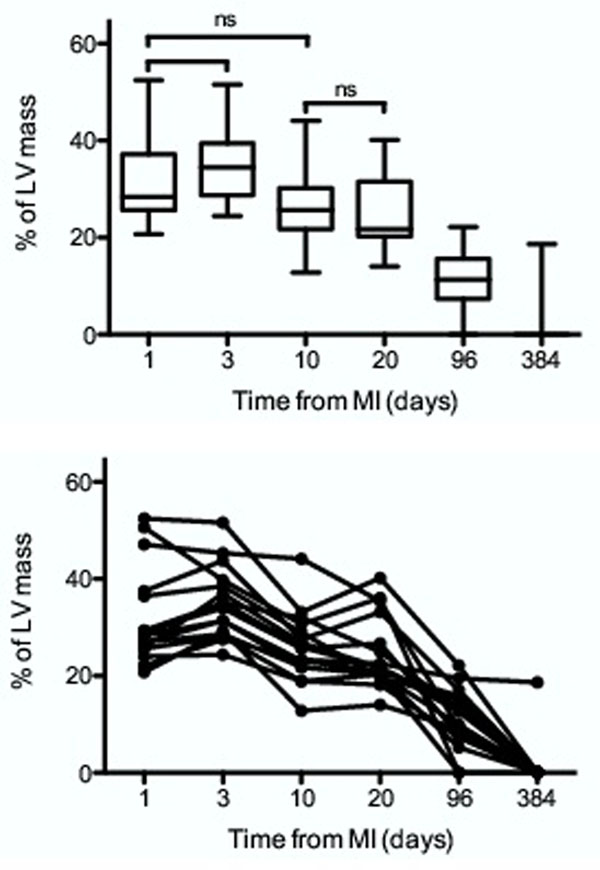
Graphs showing myocardial oedema following PPCI expressed as a percentage of left ventricular muscle mass. The top is a box and whisker plot, showing minimum 25th percentile, median, 75th percentile and maximum. Significance levels are derived from the Bonferroni's Multiple Comparison Test. Non significant differences are shown (ns) for clarity, as all other comparisons are are significant (p<0.01). The bottom shows individual observations. The x-axis is non-linear to further increase the clarity of the data.

